# Leading with local solutions to keep Yarrabah safe: a grounded theory study of an Aboriginal community-controlled health organisation’s response to COVID-19

**DOI:** 10.1186/s12913-021-06761-1

**Published:** 2021-07-23

**Authors:** Janya McCalman, Marlene Longbottom, Sara Fagan, Ruth Fagan, Suzanne Andrews, Adrian Miller

**Affiliations:** 1 Centre for Health Equity Research, School of Health, Medical and Applied Sciences, CQUniversity Australia, Lvl 2 Cairns Square, Cnr Abbott & Shields St, Qld 4870 Cairns, Australia; 2 Ngarruwan Ngadju First Peoples Health and Wellbeing Research Centre, Australian Health Services Research Institute (AHSRI), University of Wollongong, Wollongong, NSW 2522 Australia; 3 Gurriny Yealamucka Health Service Aboriginal Corporation, 1 Bukki Rd, Yarrabah, QLD 4871 Australia

**Keywords:** Self-determination, Indigenous, Capacity, Pandemic, COVID-19 response, Primary healthcare

## Abstract

**Background:**

Pandemics such as COVID-19 are a serious public health risk for Australian Aboriginal and Torres Strait Islander communities, yet primary healthcare systems are not well resourced to respond to such urgent events. At the start of the COVID-19 pandemic, a federal government advisory group recommended a rapid, tailored Indigenous response to prevent predicted high morbidity and mortality rates. This paper examines the efforts of one ACCHO, which in the absence of dedicated funding, pivoted its operations in response to COVID-19. Gurriny Yealamucka Health Service (Gurriny) is the only primary healthcare service in the discrete Indigenous community of Yarrabah, Far North Queensland.

**Methods:**

The research was conducted at the request of the Chief Executive Officer of Gurriny. Using grounded theory methods, thirteen Gurriny staff and five Yarrabah and government leaders and community members were interviewed, transcripts of these interviews and 59 documents were imported into NVIVO-12 and coded, and key concepts were compared, organised into higher order constructs, then structured into a theoretical framework.

**Results:**

Gurriny responded to COVID-19 by *leading with local solutions to keep Yarrabah safe.* Four key strategies were implemented: *managing the health service operations, realigning services, educating and supporting community,* and *working across agencies.* These strategies were enabled or hindered by five conditions: *the governance and leadership capacity of Gurriny, relying on the health taskforce, locking the door, “copping it”,* and *(not) having resources.* A year after the first case was experienced in Australia and on the eve of vaccine rollout to Indigenous communities, there have been no COVID-19 cases in Yarrabah.

**Discussion:**

The success of the locally led, holistic, comprehensive and culturally safe response of Gurriny suggests that such tailored place-based approaches to pandemics (and other health issues) are appropriate, but require dedicated resourcing. Key challenges were the fragmented and rapidly changing government processes, poorly coordinated communication and resource allocation channels, and bottlenecks in hierarchical funding approval processes.

**Conclusions:**

The COVID-19 response in Yarrabah demonstrates the need for governance reform towards greater resourcing and support for local decision making by Aboriginal community-controlled health organisations.

## Introduction

Increasing global mobility, air traffic and urbanisation have strengthened the threat of pandemics [1]. Primary healthcare services provide an effective and efficient way to improve health security and prevent or limit adverse health impacts of novel infectious diseases within particular settings through community engagement and education, rational prescribing, and implementing public health functions, including surveillance. Strengthening systems at the community and peripheral health facility level contributes to building resilience, which is critical for withstanding shocks to the health system [2]. However, relatively little attention is paid to strengthening the under-resourced primary health care systems in the places most vulnerable to pandemic diseases.

In Australia, Aboriginal community-controlled health organisations (ACCHOs) provide primary health care services to Aboriginal communities through organisations governed by an Aboriginal board and that prioritise the employment of Aboriginal staff. ACCHOs responded early to the threat of the novel coronavirus disease (COVID-19) pandemic [3]. Although pandemics are not everyday business, their vital primary healthcare role created an imperative to respond to COVID with the limited resources that they had. Throughout 2020, they prepared, prevented community transmission, delivered a response to localised outbreaks, and resumed (somewhat) normal operations [3]. Yet these responses also had implications for the organisational capacities of ACCHOs themselves, including for their leadership, staffing, financial capacity, community engagement and healthcare service delivery. Resourcing issues challenged the capacity of ACCHOs to make responsive decisions as the pandemic continued to impact Indigenous communities, both directly and indirectly.

This paper explores the learnings from Gurriny Yealamucka Health Service (Gurriny), the ACCHO that serves Australia’s largest discrete Indigenous community, Yarrabah. Yarrabah is located on the tropical north-eastern coast of Australia, 50 km south of Cairns. The CEO of Gurriny requested this research to better understand Gurriny’s organisational responses to COVID-19. Analysing and learning, in real time *during the course of* the COVID-19 pandemic (in October/November 2020) provided the rare opportunity to capture qualitative data while the effects of the pandemic were active in Yarrabah.

Pandemics such as COVID-19 are a serious public health risk for Aboriginal and Torres Strait Islander (hereafter respectfully termed Indigenous) communities in Australia [4]. Earlier pandemics (e.g. Human Influenza, H1N1) had affected Indigenous people at five or more times the rate of non-Indigenous Australians [5, 6]. High rates of chronic disease, high levels of overcrowded housing, a mistrust of authority and an ethos of emotional and physical closeness across the community were pre-existing conditions in discrete Indigenous Australian communities; these later proved to be the very conditions associated with the highest case rates of COVID-19 in UK communities [7].

At the start of the COVID-19 pandemic, it was clear that a rapid, tailored Indigenous response was needed. Based on previous studies [8–10], the Australian Government’s Aboriginal and Torres Strait Islander Advisory Group on COVID-19 recommended locally-led, holistic, comprehensive and culturally safe management of healthcare delivery [11]. ACCHOs needed to be at the centre of driving COVID-19 outbreak health measures, whilst also continuing provision of other essential primary healthcare services.

The rapid transmission of COVID-19 left ACCHOs with little time to prepare for the potential impacts of COVID-19. From the World Health Organization (WHO)’s notification of an outbreak of pneumonia of unknown cause in Wuhan city, China on 5 January 2020 it took just 20 days until the first notification of COVID-19 in Australia on 25 January, and 2 months until the WHO declared that the coronavirus disease (COVID-19) had become a pandemic (11 March 2020). Gurriny worked with other Yarrabah organisations through the Local Disaster Management Group (LDMG) which held responsibility for the disaster management response in Yarrabah. As legislated through the Queensland Disaster Management Act 2003, the LDMG reported to the District Disaster Coordination Group which reported to the State Disaster Coordination Centre. Since Far North Queensland has always been subject to flooding and cyclonic conditions, the LDMG was experienced in preparation and recovery procedures for disasters. But a pandemic was new territory and Gurriny had limited official imprimatur to influence the response.

## Methods

### Aim and design

The research aimed to examine the efforts of Gurriny Yealamucka Health Service to respond to COVID-19. Research questions were: How did Gurriny respond to COVID-19; what worked well, what didn’t, with what impact; and what learnings could be taken forward to inform responses to a potential second wave or future pandemics? A request by the Gurriny CEO to conduct the research was built on a long-term partnership between Gurriny and CQUniversity that has been culturally respectful and productive, with mutual benefit and learning. It is guided by the Indigenous Leadership Framework of CQUniversity’s Centre for Indigenous Health Equity Research, and places Indigenous priorities, end users and shared key decision-making at the centre of the research design that is respectful of different cultural worldviews, values and practices. Thus, a non-Indigenous researcher with more than 15 years’ experience in research with Yarrabah partners (JM), worked in collaboration with Indigenous researchers (ML, AM) and an Indigenous student (SF) using a constructivist grounded theory methodology, with the research approach and analysis checked iteratively with Gurriny Indigenous partners (RF, SA) [12–14]. Grounded theory studies of Aboriginal social processes fit well with the ethics of care and responsibility embedded in Aboriginal research methodologies [13].

### Setting

The traditional custodians of Yarrabah are the Gunggandji people. The community was founded as an Anglican Mission in 1892. The population has now reached approximately 4000 residents. Yarrabah was ranked in the first percentile of disadvantage in the Socio-Economic Indexes for Areas (SEIFA) index in 2016, meaning that approximately only 1% of Australia’s local government areas are more disadvantaged.

Yarrabah is located 52 km from the city centre of Cairns. Local residents travel daily between Yarrabah and Cairns to access services including schools, retail services, and employment, bringing them into contact with the domestic and international tourists that visit Cairns. This brings an increased risk of pandemic infections, such as COVID-19, to the Yarrabah community which has a large at-risk population.

As the only primary healthcare service in Yarrabah, Gurriny serves approximately 3500 regular clients. 1500 (43%) of Gurriny’s 3500 regular patients have a chronic disease, and 700 (20%) are in the extreme risk category. Chronic diseases such as respiratory, diabetes and cardiovascular diseases are known to impact COVID-19 fatality, with further risk added by multimorbidity, smoking, food insecurity, and poor access to water, sanitation, and adequate housing [15]. Previous outbreaks of infectious diseases in Yarrabah had demonstrated that the average Yarrabah housing occupancy of approximately eight people and the poor state of the existing Aboriginal housing infrastructure made quarantine measures in the community virtually impossible. Faulty taps in a third of houses also made hand hygiene measures extremely difficulty to implement (LDMG Quarantine and isolation submission, April 2020).

### Participants and documents

Eighteen people were interviewed (Table 1). Eleven participants were also Yarrabah residents living and working within the community.

**Table 1:** Participant characteristics

	Indigenous	Non-Indigenous	Female	Male	Total no.
Gurriny staff	8	5	8	5	13
Other key informants (2 leaders of other Yarrabah organisations, a Gurriny Board member, a community member and a government representative)	4	1	3	2	5
Total	12	6	11	7	18

Fifty-nine documents pertaining to the Yarrabah response to COVID-19 were also provided by Gurriny and analysed (Table 2).

**Table 2:** Documents’ characteristics

Document type	Numbers
Plans/protocols	21
LDMG meeting agendas and minutes	14
Government policy or information briefs	12
Submissions	6
Reflective documents of COVID-19 response learnings	3
Community notices	2
Department of Aboriginal and Torres Strait Islander Partnerships in collaboration with Yarrabah Aboriginal Shire Council community survey of views about the biosecurity lockdown	1
TOTAL	59

### Data collection

Participants were interviewed by one (JM) or two (JM and ML) researchers in face-to-face interviews conducted using an informal yarning method applying COVID safe research practices. Twelve interviews took place at Gurriny, four at CQUniversity and one occurred by phone. Interviews ranged from 20 to 91 min in length. Researchers provided a verbal explanation of the purpose and conduct of the research project and all participants provided informed consent.

### Analysis

The constant comparison method of grounded theory methodology [16] was applied to analyse the interview and documentary data. Starting with the documents, all data were imported into NVIVO 12 software and coded. Codes or concepts were inductively generated by asking the three generic questions recommended by Charmaz [17]:

1. What is really going on here relative to the phenomenon?
2. What concept is involved?
3. What is the basic problem faced by the participants?

**Fig. 1:**
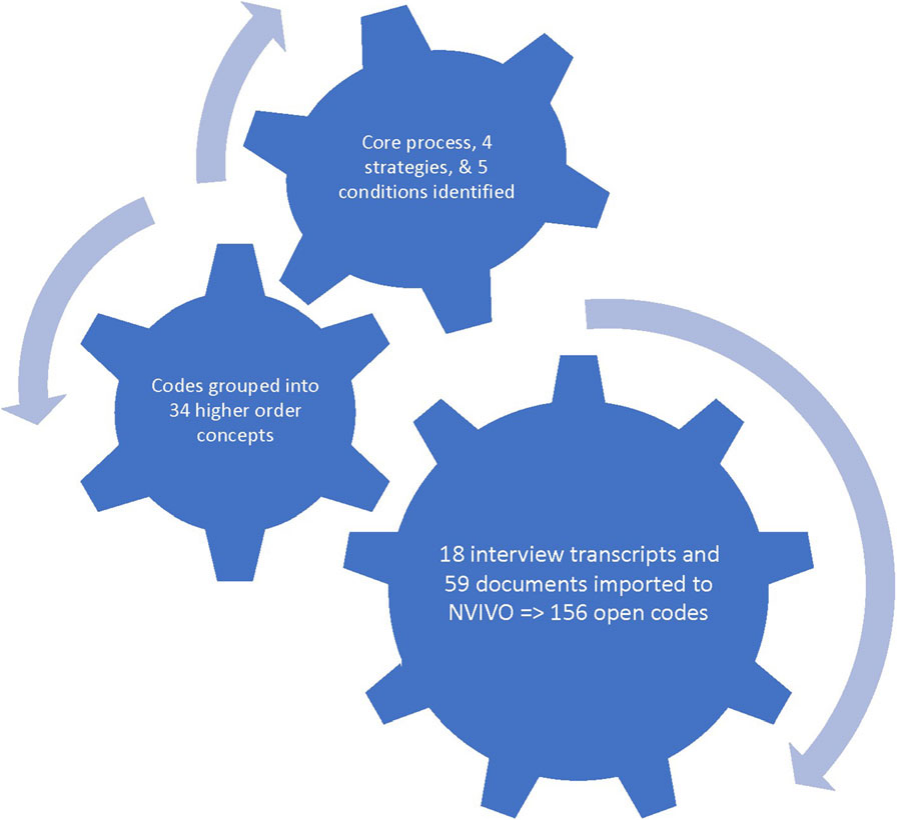
The coding process

New data from subsequent documents and transcripts were compared to existing concepts for similarities and differences. Concepts that identified events, incidents, actions and interactions that were related in meaning were grouped under higher order concepts (Fig. 1) [17].

Glaser’s causal consequence coding family [16] was then used to structure the higher order and sub-concepts into a theoretical framework that explained Gurriny’s response (Fig. 1). The framework was provided in report form for feedback from Gurriny managers, the managers of other Yarrabah organisations and to research participants who requested the research results. Feedback from a manager of a Yarrabah organisation highlighting the legislative role of the LDMG was incorporated.

## Results

### The core process: leading with local solutions to keep Yarrabah safe

Gurriny responded to COVID-19 by *leading with local solutions to keep Yarrabah safe.* At the start of the pandemic, Gurriny utilised available global mortality rate data from the developing pandemic to calculate that COVID-19 could cause a sobering additional fifty to two hundred and fifty deaths in Yarrabah in a twelve-month period. Based on these projections, the combined local health services did not have adequate equipment or trained staff to deliver the required healthcare. Decisive and evidence-based action was needed. To keep Yarrabah safe, it became evident that the first priority was to prevent entry of the virus, and if that was unsuccessful, to immediately prevent spread. A Gurriny manager considered: “*If we did have a community transmission in Yarrabah, I would hate to think how fast that transmission, and how many people would get it.”* In January 2020, a Gurriny manager presented to other Yarrabah leaders, advocating: *“It’s about local action and being decisive and leading.”*

Following an initial period of hesitation pending Queensland disaster management guidance, other Yarrabah leaders joined Gurriny in a collaborative effort. Leadership was enhanced by the pre-existing Gurriny-led Yarrabah Leaders Forum (YLF). A community leader reflected: *“We had a group of people and organisations willing to work together on the pandemic and do the best we can in terms of protecting the community*.” An LDMG funding submission for quarantine and isolation facilities (16 April 2020) recognised that: “*… local leadership and solutions with the support of resources to keep Yarrabah well and safe during this period was an important and timely message as we mobilise community to respond to this pandemic*.”

However, participants considered that the external – Federal and State Government - decision-making structures did not take adequate account of the local knowledge and capacity of Yarrabah leaders. Yarrabah community leaders were not heard in terms of what was perceived by the community to be an appropriate response. A Gurriny staff member suggested that governments should: *“get your ideas and get where the situation actually is from the local perspective …. It comes back to that old saying … , ‘please ask us what our problems are before you give us solutions.’”* Instead, local requests for resources such as personal protective equipment (PPE), and funding for a local quarantine and isolation facility, and a Communications Officer through the hierarchical state government disaster management pathway and Queensland Health decision-making processes were caught in bureaucratic bottlenecks, made more complex by the Australian biosecurity laws and processes.

**Fig. 2:**
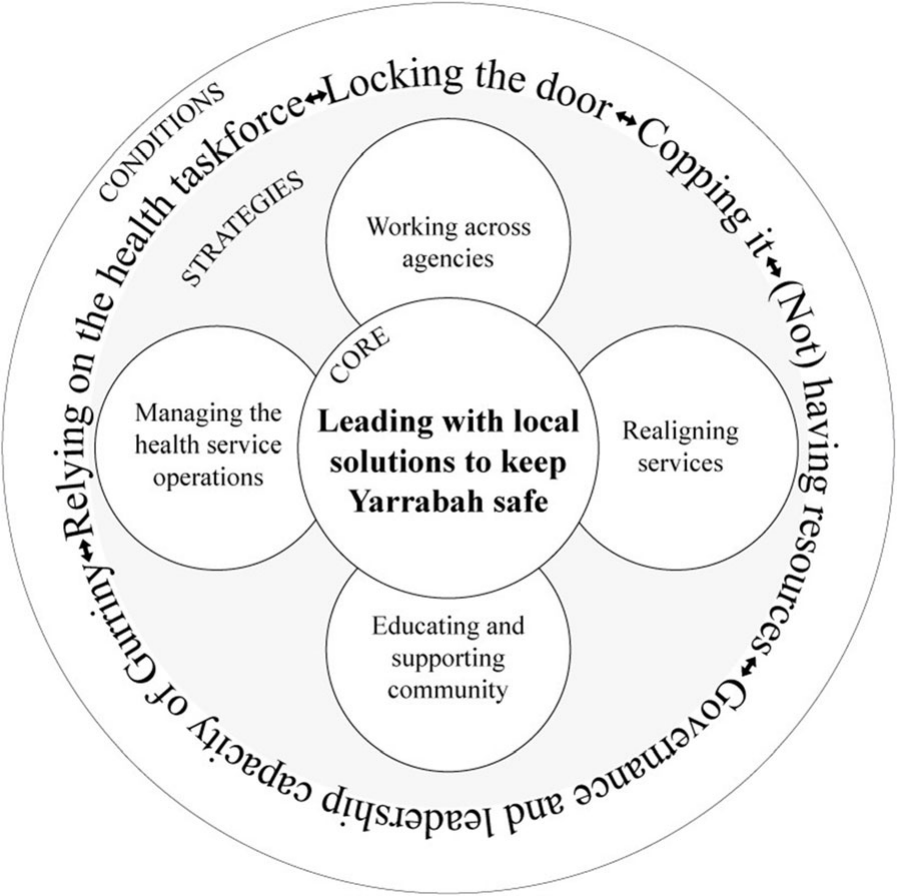
Gurriny’s response to COVID-19

Gurriny’s leadership with local solutions was at the core of its four strategies to keep Yarrabah safe: i) managing the health service operations, ii) realigning services, iii) educating and supporting community, and iv) working across agencies. These strategies were enabled or hindered by five conditions: the community-controlled governance and leadership capacity of Gurriny, relying on the health taskforce, “locking the door”, “copping it”, and (not) having resources (see Fig. 2).

### Conditions that enabled or hindered Gurriny’s response to COVID-19

The five interrelated conditions that enabled or hindered Gurriny’s response to COVID-19 are described below.

#### Community-controlled governance and leadership capacity of Gurriny

Gurriny has a holistic primary healthcare approach focused on fostering long-term generational change through family-centred programs. A Gurriny manager explained: *“We’re primary care but we’re community-controlled primary care, so we can operate in a public health space … An important part of Gurriny’s strength is their ability to pivot towards a particular problem like a public health thing, en masse”.* The holistic organisational approach enabled an agile response where the primary healthcare service quickly realigned services to reduce risk and better support clients and staff and the whole community.

Gurriny had also grown considerably in size and capacity since it assumed community control of primary healthcare services in Yarrabah from Queensland Health’s Cairns Hospital and Hinterland Health Service (CHHHS) on 1 July 2014, with finances doubling and staffing expanding from 37 to 95. A Gurriny manager reflected: “*I see ourselves as being leaders in the community … It’s a very impressive organisation.”* Another manager agreed: *“This health service is going forward in leaps and bounds and there are a lot of people watching.”* Participants commented on the strong leadership team and critical roles of the Gurriny CEO, Senior Medical Officer, and Senior Management Team (SMT): *“… having an SMT and particularly* [CEO name] *who is very receptive to well-stated points of view. You can present her with information and data and she is very quick to pick it up …*” Another Gurriny manager said*: “Our doctors were very, very proactive from day one …”* However, another Gurriny manager suggested that despite Gurriny’s leadership strength, staff members experienced a sense of fatigue in continually attempting to meet community and government expectations that were beyond the scope of their roles. It was therefore important to acknowledge the need for help, and to share the burden of responsibilities across Yarrabah organisations and State and Federal governments. She said: “*Gurriny can’t fix it all. … do we always have to feel like it’s our problem?”*

#### Relying on the health taskforce

Initially, Gurriny managers considered that the LDMG, which held responsibility for the disaster management response in Yarrabah, did not appreciate the serious health implications of COVID-19 for the community and did not provide a rapid community leadership response. A Gurriny manager noted: *“We felt that it moved really slowly, from a point of view of it being a disease outbreak.”* From the LDMG perspective, it was apparent that Gurriny did not understand the State disaster management framework. As legislated through the Queensland Disaster Management Act 2003, the LDMG reported to the District Disaster Coordination Group which reported to the State Disaster Coordination Centre. District and State coordination were managed through the Queensland Police Service. Local coordination was led by the Yarrabah Aboriginal Shire Council with the Mayor as the Chair, and representatives from ten local organisations, including Gurriny. An LDMG representative recalled: *“We had to pull them [Gurriny] up a couple of times and say, ‘ … no, no, it’s gotta come through the District because that’s the legislative framework that we’ve always been operating under* … ’ *It’s got to be controlled in an environment with a framework.”*

Resolution came with the mutual recognition by Gurriny and the LDMG of the need to work closely together. A health taskforce sub-committee was formed to advise the LDMG under the leadership of the Gurriny Senior Medical Officer and soon thereafter, the Yarrabah Leaders’ Forum (YLF) Manager. A community leader noted: *“It could’ve been different if we had a different group of leaders there. But right now, during this pandemic, the leadership that’s within the community has kept this community safe*.” A Gurriny manager suggested: *“having [YLF leader name] there and having the CEOs already used to working with each other, … you just got in and got it done … there was trust.”* The health taskforce met at least weekly from March to June 2020 and less frequently thereafter.

#### Locking the door

In late March 2020, the health taskforce of the LDMG agreed that there was clearly a need to lock down Yarrabah to prevent the spread of COVID-19 from Cairns. A Gurriny staff member reflected: *“I would always say that was the best thing that Yarrabah did because we didn’t see no cases in Yarrabah …”* Leaders recognised that residents needed a few days to prepare for a lockdown to stock up on food and household items. While leaders were notifying Yarrabah residents that they had a three-day grace period to get prepared, however, the Australian government pre-empted the local initiative by announcing a Human Biosecurity Emergency Determination. A Gurriny staff member reflected: *“We were saying, ‘well look at least we need two or three days’ … We were halfway through the meeting minutes, someone goes, ‘I just got a text from someone and the police are setting up checkpoint on the range right now’ …”.*

The determination was introduced in Yarrabah at midnight on 25 March 2020, effectively locking down the community immediately for almost 3 months. Access was restricted by road and sea, with the police and army providing 24/7 staffing of a checkpoint by road, and border security monitoring sea travel. A community leader recalled: *“People weren’t prepared. People didn’t have the time. They didn’t have any money that week.”* Consistent with government health guidelines, community members were told that they would be required to undergo a 14-day minimum period of isolation before entry or re-entry into Yarrabah. Exemptions were permitted for essential activities or in urgent circumstances. Lists of essential services and essential workers were compiled, but the criteria and application forms changed frequently, creating additional confusion. A Gurriny manager reflected: *“I don’t think the rules got communicated well enough. But I think that they didn’t get communicated well enough because there was too much going on.”*

The checkpoint was patrolled by state police and federal army officers, and became a contested space. Gurriny staff worried about the maintenance of infection control procedures. A staff member commented: *“The police at the lockdown at the gate to the community were doing things that didn’t make sense … they were letting people through who shouldn’t have been let through.”* Multiple and varied responses to requests at the checkpoint led a Gurriny staff member to comment: *“prevention relies on a checkpoint that was dysfunctional at best”.*

Breaches of the lockdown rules by walking track and boat were also of concern. Since the lockdown cut the supply of alcohol, community members attempted to access alcohol via bush tracks, later dubbed “the COVID Highway”. There was concern that those using the ‘Highway’ were not only putting themselves in danger and breaching Yarrabah travel and access restrictions, but also putting community members at risk. A Gurriny manager noted: *“There was two tracks … bringing in drugs and alcohol.”* Fishing was initially banned; later fishing at the beach or in the bay was allowed if the COVID-19 rules were followed. A Gurriny staff member commented: *“And they did stop traditional hunting as well – with the boats... That was where stress come from because people were- they can’t go out to Cairns to get food, but they can’t go do traditional fishing or hunting where they can support families.”* A Gurriny manager also suggested that such rules could potentially have been more accommodating.

By 26 May, after 2 months of lockdown, an LDMG submission noted that other Queenslanders were being permitted to travel outside their suburbs and move back to a normal lifestyle. A community leader reflected: *“when the Premier announced a lot of the easing of [Queensland] restrictions, most of our discrete communities weren’t included in that because we came under the Biosecurity Act. It was tough …”* This was a challenging time as there was constant confusion in regards to whether the blanket Commonwealth biosecurity legislation or more nuanced Queensland Public Health legislation applied at any particular time. For example, as the restrictions were eased, the rules changed frequently and became complex and hard to follow, thus prompting the LDMG to develop a Roadmap. A Gurriny manager recalled: *“A lot of the … people who live in Cairns who wanted to go and see family thought that that meant they could go over. And then they couldn’t go over, so then they were a bit cranky.”* The Yarrabah lockdown was lifted on 18 June, 2020 by Federal Minister Greg Hunt. A Gurriny manager described celebrating the ending of restrictions: *“Just … leaving for Cairns was unreal … that drive from here to Cairns … is what helps me with some of my self-care.”*

#### Copping it

Most community members recognised that the lockdown had been imposed to keep Yarrabah safe. But community members were concerned about the practical implications. A Gurriny manager recalled: *“No one liked the lockdown because of the inconvenience.”* Another Gurriny manager said: “*I think they really couldn’t understand … why we weren’t allowed to go up to Edmonton …”* . Edmonton is a 30 min drive from Yarrabah and offers access to vital necessities such as groceries and fuel.

For some community members, deeply-held memories of historical disempowerment were triggered, along with responses to contemporary racism and discrimination. A Gurriny manager explained: *“When you start saying we’re gonna lock you down to protect you, that’s quite paternalistic ... You could only really expect people to accept it if you can explain it …”* Another Gurriny manager recalled: *“It was a first time in my journey in life as an Aboriginal man that I felt the power of the Crown yeah … I felt the force- this power that exists over our nation and over our people. Just being exercised at a drop of a hat!”* This reminded some community members of the former years of being under the Aboriginals Protection and Restriction of the Sale of Opium Act under which reserves were established to which Aboriginal people could be forcibly relocated, with basic civil rights removed. The psychological effect of the lockdown had particular impact on Elders. A Gurriny manager suggested: *“A lot of the elderly groups and the ones that were living through the exemption time, had triggered back from that time where you had to hand in your papers to go to and from community.”* A community leader commented: *“there was like a revisiting old issues you know, and being restricted and locked up … like the old days you know, in the dormitory.”*

The frustration of some community members with the lockdown was directed at community leaders and created community divisiveness at a time when they were already struggling to manage a range of challenging issues. A community leader reflected: *“There was a group within the community that opposed everything that was going on … leaders in the community were being attacked either on social media, or in person …”* Another community leader said: *“We copped it there for a good couple of months … We’ve had to be diligent about our efforts but also take the hits too for it!”* The frustration of community members increased over time, culminating in protests by people in the community wanting to leave Yarrabah and by people in Cairns wanting to enter the community. Dubbing this *‘protest in, protest out’* the leader of a local community organisation explained: *“They wanted to come back on Country – visit family … While the other mob was getting a bit tired of being locked down …”.*

Some Gurriny staff members were sympathetic to the frustrations. A manager said: “*Like, a lot of us at Gurriny had great empathy for the protests and anger …” However,* another Gurriny manager said: *“I think sometimes some people felt … it was all about me as an individual rather than a whole [community].”*

However, the protests began to directly threaten the safety of Gurriny staff. A Gurriny staff member described: *“We were lucky - quite supported by our CEO. The moment we had protests coming towards the clinic for the lockdown, because they were aimed at us being seen as the face of the lockdown um … our CEO shut us down, sent us home.”* A community leader commented: *“They put on the victimhood mentality around the history and the impact of colonisation on our mob ... we need agitators but I think their agitation is being stuck on not moving forward …”* External media provided coverage of the protesters’ cause and added fuel to the protests.

#### (Not) having resources

Yarrabah did not have the needed human, financial and protective resources to respond adequately to the COVID-19 threat. A Gurriny manager suggested that resourcing shortages were a result of a Federal government failure to resource the response: *“It’s cost the State Government an enormous amount of money to do that. That was the beginning of the end.”* Participants considered that even State-funded public health services had inadequate resources to manage the pandemic response. A Gurriny manager identified: “*It’s a Public Health emergency … I think there’s a veneer of capability to deal with it. … The hospital’s not ready for it. The local Tropical Public Health Service (TPHS) are trying their best, but they’re working with nothing, with limited resources …”.*

Submissions through the health taskforce of the LDMG were progressed to trigger the provision PPE, a local quarantine and isolation facility, and a Communications Officer, but deficiencies in understanding the process and pathways for escalating communication flows from the LDMG, through the DDMG and then to the state level under the recently amended Queensland Disaster Management Act (2003) created ongoing delays in decisions*.* A community leader recalled*: “We had a lot of requests for assistance ... we never got communications back … it was just constantly trying to find out well where is it? Who’s actually responsible? Can we get some feedback as to where that process might be? It seemed to be quite disjointed.”*

Funding for Gurriny was also an issue. A reduction in patient visits in the early stages of the pandemic had meant a 50% drop in Medicare income, the main source of untied funds received by the organisation. The saving grace was the organisation’s eligibility for and receipt of Job Keeper subsidies to maintain staff salaries. As noted by a Gurriny manager: *“We’re not funded from public health, we were not funded for any of this pandemic until later on in the piece when funding started to trickle in.”* Responses to Gurriny’s requests for resources were also slow.

As the biosecurity restrictions eased, State Government resources were provided for public health services. As a Gurriny manager recounted: “*The Public Health Unit has now received additional funding, so they’re actually expanding their team … I feel a lot more confident that if we go back down this track that we’ve got things in place.”* However, at the time of interview in November 2020, funding through the disaster management pathway remained problematic.

### Strategies that Gurriny implemented

At the start of the pandemic, Yarrabah had insufficient trained public health workers, testing facilities, isolation or quarantine facilities, PPE, and ventilators for the potentially large number of high-risk COVID-19 patients. The strategies undertaken by Gurriny in response to these early scenarios can be broadly categorised into four interrelated types: managing the health service operations, realigning service delivery, educating and supporting the community, and working across agencies.

#### Managing the health service operations

The four key aspects to managing the Gurriny operational response to the pandemic were being informed and prepared, working as a team, establishing a Cairns-based Yarrabah hub, and looking after staff.

As early as November 2019, Gurriny’s managers were aware of the global pandemic threat. A Gurriny manager recalled: “*probably November last year … we were paying attention to what was happening in China … just monitoring it … There didn’t seem to be a lot of Federal level movement on anything … so we watched what was going on essentially.”* However, Yarrabah’s preparation was hampered by the rapidity of change in information about the threat and a lack of specificity to the local circumstances. A Gurriny manager said: “*The information that was coming through to the LDMG … was very broad, like ‘these are the restrictions’ or ‘this is what we’re doing’ … we had … a heightened sense of anxiety that we needed to move a lot quicker around this …”.*

Gurriny had previously experienced infectious disease outbreaks in Yarrabah and had learnt how to effectively manage them. A senior Gurriny manager reflected: “*There’s been three public health emergencies … really helped prepare … So, we were able to highlight the concern very quickly, make an argument for that pivoting again …”.*

Gurriny also had a Quality Assurance Officer and procedures in place to deal with disasters; this expertise was foundational for working through LDMG pathways to effect a pandemic response. A Gurriny manager commented: *“There is some element of pandemic disaster planning that’s within the accreditation cycle generally but … I don’t think anyone really understood the scope and the size of an actual proper pandemic.”*

The organisational capacity of Gurriny was constrained during the COVID-19 lockdown by workforce continuity issues. Gurriny’s workforce comprises 75% local Yarrabah staff but there is also a heavy reliance on external agency staff. With lockdown restrictions, exemption permits were required for this skilled workforce to enter and exit Yarrabah and some staff members chose to not continue employment. A Gurriny manager explained: *“people are obviously going to put their own needs first … So we lost some staff.”* Gurriny managers were also concerned to protect staff members who also had underlying chronic conditions. A manager noted: *“Some of our staff in Gurriny also fitted into that ‘at risk’ category as well and so the balancing of not putting them at risk versus helping them to maintain employment.”*

In part due to such shortages, staff were assigned new roles and responsibilities. A team-based approach was supported and the capacity of staff members grew. Opportunities were provided for staff members to step up into opportunities of new leadership positions. One manager reflected: *“I was able to exercise some of my skills if you like, in a way that I felt contributed to some of the promotion and in the community around knowledge sharing …”* A staff member said: *“I’ve had huge professional growth within the last six months”.* Following lockdown, Gurriny took on additional staff to fill vacant positions, such that at the time of this study, a manager observed: *“We’ve had complete staff turnaround before, during and after.”*

A creative solution for maintaining the continuity of employment for Gurriny staff who lived in Cairns and were thus locked out of Yarrabah was to open a Cairns-based Yarrabah outreach clinic. It was established at the Cairns-based ACCHO, Wu Chopperen Health Service, which generously volunteered the use of office space. The clinic was useful also for providing care for Yarrabah patients that needed to travel to Cairns for hospitalisation or other treatment. A manager described it thus: *“There were a number of a staff who were able to be a part of that team and it’s that whole idea of redeploying.”* A Gurriny manager recalled: *“we set up an outreach service in Cairns that supported our mob in Cairns that weren’t able to come back to community or were in quarantine for the fourteen days …”.*

The clinic became intensely busy, supporting patients with multiple needs. A manager recalled: *“There were many people ‘caught’ for want of a better word … we supported them during that time …”* A considerable focus of the Cairns-based clinic entailed supporting patients to complete paperwork to allow exemption from the biosecurity restrictions and permission to enter Yarrabah. A Gurriny manager recalled: *“The way that they did the passes and the forms for that checkpoint changed constantly ... In one day alone, the forms changed five times … in terms of having to provide emergency care for a critically ill patient … it can be quite stressful and frustrating* …” In summary, a Gurriny manager considered*: “If we didn’t have that [Cairns-based] team at the time, it would’ve just been really difficult.”*

Staff members became fatigued through dealing with the realignment of roles and intensity and complexity of the needed COVID-19 response. A Gurriny staff member recalled: *“We’d work all night to get process, get communications in place and make sure that we were ready by the next day to give the best response possible …”* Yarrabah-based staff experienced the difficulties of separating work from the impacts of the lockdown on family life. A manager explained: *“I think we need to understand that if we’re not looking after ourself then we can’t look after others … We do need to have some awareness around the fact that if you’re a community person or professional, you never go away. Work never ends.”* Those living in Cairns and working from home experienced different impacts of disconnection and isolation. The Gurriny CEO recognised the fatigue of staff members and responded by instigating generous support measures.

However, at the end of the lockdown, many Gurriny staff and managers were exhausted and took much needed leave. A manager recalled: *“I actually took three weeks ... For the first week, I actually considered whether I needed to go and get counselling to debrief because it had been so intense …”* A Gurriny manager suggested a need for further internal organisational communication to protect staff wellbeing: *“I think we need to have regular briefings and I think we need to start listening to one another.”*

#### Realigning services

Service delivery provision was radically extended, with realignments made to patient care; testing; infection control; the “sterile tunnel” whereby patients were permitted to travel to Cairns for short-term medical appointments so long as they did not divert from their intended medical destination or require an overnight stay, and Cairns quarantine; and isolation requirements and facilities.

Service delivery realignment occurred organically through a cyclical process responding to service needs that changed over time. At the start of the lockdown, regular Yarrabah patients who were isolating at home were advised not to come into the clinic unless necessary. A manager recalled: *“We definitely saw a restriction of services, like everything contracted to core services.”* A Gurriny manager added: *“There was a radical change. So a lot of our program activities stopped … A lot of the medical appointments … in terms of all the visiting services that went into Yarrabah were cancelled.”*

The realignment of services was accompanied by a need for funding flexibility and tracking of COVID expenditure. A manager recalled: “*Straight away in March I said to* [finance manager name] *‘We don’t have any money. Let’s just set up the line item should money come later than we can just … pay it back.’”* Gurriny contacted their funders and advised of the need for flexibility to use existing funds for the management of the pandemic in Yarrabah.

Just prior to the biosecurity lockdown at Yarrabah, a fever clinic was established in the undercover entrance outside the co-located Gurriny/CHHHS clinic building, to test community members for COVID-19. Although the fever clinic was the responsibility of CHHHS, Gurriny agreed to contribute. A Gurriny manager recalled: *“We need to protect the staff and we need to protect the vulnerable patients who come and see us for valid reasons because we still need to run primary care.”* A staff member noted: *“It was quite physically hard on our staff, especially in the beginning of the year when it was in thirty-six degrees and you’re sweating … in the beginning we had full PPE, and staying out there for the whole shift, and that was just so exhausting.”*

There were complexities in running the fever clinic between two organisations, and in supplying the appropriate equipment and protocols. A Gurriny manager explained: *“I spent a fair bit of time making sure that if we were going to take on testing, that we were going to do it right …”* Establishing the fever clinic meant that the physical Gurriny clinic layout needed to be altered, staff needed to be trained, and clients required education as well as testing. A Gurriny staff member estimated: *“We did about four per cent of the population within the first month or something.”*

By November 2020, Gurriny handed back responsibility for the fever clinic to Cairns and Hinterland Hospital and Health Service (CHHHS). The focus shifted to working with CHHHS and trained Queensland Health public health staff for preparedness for contact tracing in readiness for potential cases of COVID-19 A staff member commented*: “You need a local person from community to be able to assist in that as to where are the family groups, who knows who and who would have gone where.”* Another Gurriny manager described the planning process: *“We’re in the process now of developing up a procedures document as part of our Rapid Response Plan … if somebody in Yarrabah was made positive, what would happen to that patient? And then what would happen to their family? So we need to have that information out there.”*

At the start of 2020, Gurriny revised their emergency management plan and infection control procedure. Due to global shortages of PPE and a lack of clarity about distribution mechanisms, it became difficult to access PPE. A Gurriny manager recalled: *“The Commonwealth was providing PPE to the Primary Health Network … we got one box of fifty or a hundred masks I think it was, and that was all we got … I mean I don’t know what would’ve happened if we had a case.”* Supplies of PPE were ultimately obtained through a media appeal from a U.S.-based supplier.

Gurriny undertook basic training of staff and patients in infection control but identified a need for further and ongoing training. Gurriny also offered to provide infection control training to the Police at the road checkpoints, but the offer was not taken up. A Gurriny manager commented: *“It’s the same with like in the quarantine hotels in Sydney and Melbourne – they need medical people making those decisions. And training the staff and teaching them, ‘no you can’t touch that. You can’t do that.’”*

As Gurriny began testing people through the fever clinic in late March 2020, the absence of a quarantine site meant that those awaiting their results were sent home, despite the overcrowding in Yarrabah houses. A Gurriny staff member commented on the requirement for a four-day quarantine period: *“The quarantine information was constantly changing as well. So it was like whether they had to quarantine, or the entire house had to quarantine. That’s changed a couple of times … it just depends on the Queensland Government’s … instruction.”*

The health taskforce of the LDMG investigated the potential for existing infrastructure to be used for quarantine and isolation purposes in Yarrabah, along with the need for appropriate fit out, access to amenities and support staff. Three sites were identified as potentially suitable. However, by mid-April, the lack of forthcoming funding for a quarantine site/s, and concern about staff risk, organisational liability, not having enough PPE and the complexity of decontamination and waste management, caused the health taskforce to revise the plan to put more onus on families to look after themselves. The revised plan encompassed a multi-pronged approach, including home isolation with the necessary support, and the development of a quarantine site for use if people needed to get away from their homes. A Gurriny manager considered, however, that there was insufficient capacity in Yarrabah to manage an outbreak of COVID-19. He said: *“Realistically the only people who can do that are the government, and through the armed forces - the medical armed forces.”*

The lockdown of Yarrabah significantly impacted Gurriny’s patients who were accessing treatment in Cairns, including for short-term medical appointments and longer-term hospitalisation. In accordance with the lockdown rules, those entering Yarrabah needed to have completed 14-days of quarantine in an approved facility. As a Gurriny manager recalled: *“We were able to have agreement around the sterile tunnel so that if there were people who urgently required certain appointments, we could bring them in and then bring them back home.”* However, as a Gurriny staff member described: *“How it actually operates on the day depends on staff on the floor and whether they’ve been told or whether they have capacity to separate people.”*

If a Yarrabah person required overnight hospital accommodation, they needed to abide by the 14-day quarantine period upon discharge before returning to the community. A Gurriny manager explained: *“They had to do the fourteen days, but they couldn’t do fourteen days until they were medically cleared.”* The information and preparation provided by CHHHS to patients before exiting Yarrabah was often inadequate. A Gurriny manager explained: *“There was a bit of a conversation at the A&E [CHHHS Accident and Emergency] at Yarrabah to say, ‘look once you go in, you’re gonna have to do your fourteen-day iso [isolation]’, but nobody really explains that on average, you’re probably going to be away from home for at least three weeks minimum … usually they get in there, it’s an emergency so they don’t have their clothes, they don’t have their money. Then they can’t see their family members so then they want their family members to come in …”* Keeping track of more than 100 patients, their support people and families through their journeys from Yarrabah to hospital, to quarantine, and back to Yarrabah was challenging. Gurriny worked with CHHHS Nurse Navigators to track patients- a role well outside its normal scope of operations.

#### Educating and supporting the community

The two aspects of educating and supporting the community were relaying the COVID-19 message, and supporting the wellbeing of community members, including their food security, mental health, alcohol and drugs, funerals, and youth wellbeing.

From January 2020, prior to lockdown, Gurriny started to consistently relay COVID-19 educational messaging to community members through Facebook and other social media. A Gurriny manager recalled: *“We produced a series of posts and videos talking about increasing people’s hygiene, handwashing, surface cleaning with bleach and all that sort of stuff which was really important in those early stages. We couched this within a message of protecting your mob.”* Gurriny managers considered their use of social media had been effective in spreading the message.

By the start of the Biosecurity Act Determination lockdown on 26 March, the LDMG health taskforce had agreed that clear communications to community members must be high priority. However, the LDMG and Gurriny leaders acknowledged that they did not have the required resources or established systems to effectively communicate COVID information about the biosecurity determination, the rules of lockdown, and what they could expect in terms of length of the lockdown period to Yarrabah residents. A manager commented: “*None of the services at the time had a dedicated Communications Officer who could get different forms of messaging and coordinate that.”*

At the start of April, Gurriny and Gindaja (Alcohol Rehabilitation Service) teams commenced the resource-intensive initiative of door knocking with pamphlets through house-to-house visits to get the COVID-19 prevention messages out. Gurriny staff were trained internally. A Gurriny manager recalled: *“We kind of brought the clinical and the health promotion, the SEWB all together … we handed out resources, we did information sharing, we talked about services that was provided, people who they could access … how to present for medication.”*

Household visits also provided Gurriny with community feedback. A Gurriny manager observed: *“… something that I heard a lot of when I was out … was ‘why were we in lockdown?’ And ‘why were people that didn’t live here coming through?’”* Two community surveys were also conducted to gauge community opinion about potential lifting of the Commonwealth Biosecurity Restrictions, and a potential amendment of Alcohol Management Plan restrictions.

Later, as the lockdown restrictions were being eased, the LDMG health taskforce emphasised the focus on householders’ responsibilities to care for their own safety. A Gurriny manager explained: *“We’d help them work through a plan and we’ve talked about also providing a starter pack … like sanitising and things like that. But it also has a part of it which is where you can as a family, make some decisions ...”*

While the primary effort of Gurriny was rightly focused on protecting Yarrabah community members from COVID-19 infection, the community lockdown under the biosecurity regulations also aggravated extant health and social issues. In particular, it affected the security of the food supply and created challenges related to the truncated supply of alcohol and drugs, altered funeral arrangements, and the need to support the wellbeing of community members during uncertain and frightening times. The role of Gurriny was described by a manager as being to: *“reassure and encourage people they had to be part of that process. It wasn’t just something that we could fix.”*

The lockdown interrupted the normal shopping routines of Yarrabah residents at Cairns supermarkets. To enable community closure, the LDMG needed to ensure alternative arrangements for food supply. A Gurriny manager recalled the supportive role played by the Gurriny health promotion team: *“I know the Health Promotion Team … helped Council with the Woolworths and Coles stuff … they gave people reassurance.”*

COVID-19 and the community lockdown brought a range of challenges to community members’ wellbeing and mental health. A Gurriny manager described: *“The stress I saw in the early days was the unknown … people just needed to be reassured that it wasn’t only happening in Yarrabah; it wasn’t only happening in Australia; it was happening world-wide.”* Gurriny staff reported community members and Gurriny staff experiencing elevated fear, anxiety, bereavement and trauma. Gurriny had ceased conducting their group wellbeing programs due to social distancing requirements. Instead, the social and emotional wellbeing team maintained the delivery of wellbeing and mental health services through home visits and other one-on-one and family support.

Yarrabah has an alcohol management plan that restricts the amount of alcohol entering the community, but lockdown completely stopped the legal supply of alcohol and drugs. Yarrabah residents who live with an addiction began to present at A&E with alcohol-related withdrawal issues. An appetite for sly grog (illegally sold alcohol) was fuelled in part by additional funds coming into the community through government Job Seeker (unemployment benefit) payments which had been temporarily supplemented to mitigate the early impact of COVID-19. An alcohol working group of the LDMG was established to look at issues relating to alcohol access. By mid-April, it was agreed that a harm minimisation approach should be taken, and the committee examined options such as a licensed venue operating with a focus on social reinvestment.

A Yarrabah funeral safety plan was drafted by the LDMG in June 2020. The plan was based on government guidelines for a COVID-safe funeral, including maximum participants and introducing technology to stream services and prevent crowds gathering. However, a Gurriny staff member recollected: *“Sometimes cultural behaviours will surpass Government direction or infection control desires ...”*

Youth wellbeing programs and the availability of the youth hub space were casualties of the service delivery realignment and requirements for social distancing. This meant that: *“we didn’t do much for the youths, but we couldn’t as well” (Gurriny staff member).* A Gurriny staff member commented: *“Yarrabah was in lockdown but there were still things to do as well. We did see a resilience in family going to beaches … fishing off the beach or camping”.*

With the Queensland Government closing schools and implementing home-based learning for the first 5 weeks of term two, children and young people in the community were left to the care of their families and/or their own devices. However, a particular subset of youth was strongly impacted. A Gurriny staff member noted: “*School leavers … there were a lot of stress on getting back to school.”* Furthermore, the 202 Yarrabah students who attend 12 boarding or day schools outside of the community did not have a dedicated space to learn and were working with limited access to Wi-Fi/internet to connect to their school. Gurriny provided space and computer facilities in a role that was clearly beyond their scope.

#### Working across agencies

Gurriny worked closely with other Yarrabah-based NGOs and government agencies, and with government agencies outside Yarrabah. Collaboration with Yarrabah organisations strengthened relationships. For example, a Gurriny manager said: *“We had Gindaja with us. We had Council work with us. They came from the Day Care and different areas. From housing. I think that was really positive. I think that’s what the community needed to see at the time … their mob was doing something.”*

Relationships with government bodies located outside of Yarrabah were reported as less collegial. For example, Gurriny staff expressed frustration at their unsuccessful attempts to influence Police infection control practices at the checkpoint. Intersectoral communications through the disaster management pathway were also problematic. A Gurriny staff member identified the need for: *“Succinct, actual communication pathways and ensuring that the specific roles … really need to be embedded.”* Different organisational systems created communication problems. A Gurriny manager suggested: *“If you had a common thread all the way through, it was communications at the end of the day which was actually the problem …”* Communication issues between Gurriny and other organisations were difficult to resolve and often required escalation to high levels of government. A Gurriny staff member observed: *“Our CEO is in contact with all the big-wigs and was able to get us information quicker than what we could get through official channels with CHHHS or Queensland Health …”* Towards the end of 2020, Gurriny managers were making headway in strengthening relationships with some departments.

### Impacts

Gurriny responded to COVID-19 by *leading with local solutions to keep Yarrabah safe*. Undisputedly, the major benefit of their efforts was that Yarrabah succeeded in preventing cases of COVID-19 coming into the community, thus preventing potentially devastating morbidity and mortality*.*

Participants acknowledged that although challenging, many things worked well regarding the COVID-19 response by Gurriny and the Yarrabah community. The community-controlled governance and leadership capacity of Gurriny enabled the primary healthcare service to quickly extend the nature and reach of its operations and radically realign its holistic service delivery model to prevent COVID-19 transmission. Group-based programs were postponed and instead, Gurriny utilised social media and committed to an ambitious community door knocking effort to provide COVID-19 information and advise the 4000 community members about food supply options, hygiene, social distancing and other preventive measures. An LDMG submission to amend the biosecurity measures (26 May 2020) acknowledged: *“This could not have been done without the community listening and doing what was asked.”* An effective fever clinic was established at the clinic with up to 500 residents tested. Gurriny upgraded its infection control procedures and trained staff, identified and established local quarantine facilities, and managed the patient journeys of more than 500 residents to attend specialist medical appointments or hospital in Cairns and back to the community throughout the designated biosecurity lockdown (26 March to 18 June, 2020). These healthcare processes are now familiar to Gurriny staff and Yarrabah residents, and will serve the community well in the event of a second wave or further pandemic.

Through the LDMG health taskforce, relationships between Yarrabah organisations and engagement with some community members were strengthened. Unintended benefits of the lockdown included operationalisation of the CHHHS Yarrabah dialysis service to avoid unnecessary travel for renal patients who had previously travelled to Cairns. A manager reflected: “*Previous to COVID, we’d been fighting to have dialysis full service in Yarrabah rather than driving our mob into Cairns every day … COVID forced that …”* Similarly, an isolation and quarantine facility is planned for construction at Gindaja. By working together as a team, Gurriny and other organisations recognised their enhanced capacity to achieve “*their best for the community and the best for Gurriny” (Gurriny manager).*

Many of the things that did not work so well about Yarrabah’s COVID-19 response resulted from government failures to respond adequately to Yarrabah’s requests for information, support, and timely resourcing for needed equipment, facilities and trained personnel. There was acknowledgement that the Federal Government’s community lockdown and the Queensland Government initiative to close the state borders contributed to keeping Yarrabah COVID-19-free. A manager said: “*I don’t think it was our contribution alone.”* However, implementation was problematic, with the rules for the lockdown being unclear and changing constantly. As a result: “*people just didn’t understand ‘this is a rule’. It’s not a suggestion, it’s an actual rule that has consequences” (Gurriny manager).* The multi-jurisdictional nature of the LDMG and blockages in decision making pathways created ongoing delays in resourcing decisions such as for vital PPE, local quarantine and isolation facilities, and a Communications Officer position. The resultant resource shortages created unnecessary stress and fatigue for Gurriny staff, and insecurity and frustration for community members. Community frustrations caused divisiveness, stress for leaders, and a threat to Gurriny staff safety. There are also predicted longer term exacerbated impacts on community members’ mental health.

Gurriny continues to demonstrate leadership by preparing for a second wave of COVID-19 and future pandemics through strengthening its communications, youth wellbeing programs, training and preparation for infection control, contact tracing and case management, and through the establishment of local isolation and quarantine facilities and a vaccination program. A Rapid Response Team and Plan have been established to progress such issues. A manager reflected: *“I’m not sure what community people are thinking. I think they think it’s ended and we’re all good, but from Gurriny’s perspective, it’s not over yet.”* Yarrabah has not yet taken the time to debrief or celebrate its successes and achievements in maintaining the safety of its community and residents through COVID-19. A Gurriny staff member considered: “*If you never have to deal with it well … what we did put in place has worked.*”

## Discussion

Australian policy makers and academics predicted that if infected with COVID-19, high levels of chronic disease and socio-economic inequities would place Indigenous Australians at a heightened risk of mortality [15]. This prediction has indeed been realised in Indigenous populations in other countries [15]. This paper examined the response of one ACCHO to prevent exposure to COVID-19 in its community, and hence prevent such mortality. Gurriny responded to COVID-19 by *leading with local solutions to keep Yarrabah safe*. A year after the first case was experienced in Australia and on the eve of vaccine rollout to Indigenous communities nation-wide, fewer than 1% of Australia’s 29,037 cases of COVID-19 were Indigenous, and no infections had been experience by Yarrabah people [18]. This Yarrabah result supports the recommendation of the Australian Aboriginal and Torres Strait Islander Advisory Group on COVID-19, that acknowledged the capability of ACCHOs to provide locally-led, holistic, comprehensive and culturally safe management of healthcare delivery [11].

The findings that local Indigenous ownership and control was critical to the effective response to COVID-19 supports and extends broader evidence for community ownership of governance and improvement processes [19, 20]. Tailored, place-based approaches to pandemics (and other health issues) allow for evolving governance processes, relationships, institutions, and structures [21]. In turn, these enable rapid, extensive and culturally appropriate responses to health service operations, realignment of service delivery, education and support of the community, and strengthening of partnerships.

The alliance of Yarrabah organisations through the LDMG health taskforce provided a creative local solution to interfacing with the hierarchical legislated disaster management response pathway. However, obstructions to Yarrabah solutions caused by the top-down, blanket directive to “lock the door”, fragmented and rapidly changing government processes, poorly coordinated communication and resource allocation channels, and bottlenecks in hierarchical funding approval processes [19, 20] demonstrated the need for governance reform towards greater resourcing and support for local decision making. Such reform is consistent with the hypothesis that health equity will not be achieved without dramatic redistribution of power and resources [22].

COVID-19 is predicted to persist through several years of a halting post-pandemic transition marked by ongoing viral evolution, localised outbreaks, and possibly multiple rounds of updated vaccinations [23]. At the time of writing this paper, Gurriny is preparing to administer the recently approved Pfizer/BioNTech and University of Oxford/AstraZeneca COVID-19 vaccines which are prioritised for roll out to Indigenous Australian adults through ACCHOs, other primary healthcare services and pharmacies [18]. The effectiveness of Gurriny’s vaccination effort is likely to depend on the extent to which local organisations can overcome the practical constraints of vaccination, address potential community concerns and promote a sense of community cohesion. In the context of a potential opening of international borders, including the port of Cairns, Gurriny’s *leadership with local solutions* will continue to play the central role in *keeping Yarrabah safe* into the future.

## Data Availability

The datasets generated and/or analysed during the current study are not publicly available due to potential that transcripts of interviews with participants may lead to their identification (given the small pool of Yarrabah leaders) but are available from the corresponding author on reasonable request and with permission of Gurriny Yealamucka Health Service.

## Abbreviations

COVID-19: Corona Virus disease, 2019
ACCHOs: Aboriginal Community Controlled Health Organisations
Gurriny: Gurriny Yealamucka Health Service Aboriginal Corporation, Yarrabah
H1N1: a subtype of the type A influenza virus, distinguished by a mutation of hemagglutinin (H1) and a mutation of a neuraminidase (N1)
WHO: World Health Organization
LDMG: Local Disaster Management Group
SEIFA: Socio-Economic Indexes for Areas
YLF: Yarrabah Leaders’ Forum
CHHHS: Cairns and Hinterland Hospital and Health Service
SMT: Senior Management Team
TPHS: Tropical Public Health Service, Queensland Health
PPE: Personal protective equipment
